# Skeletal Muscle Development in Postnatal Beef Cattle Resulting from Maternal Protein Restriction during Mid-Gestation

**DOI:** 10.3390/ani11030860

**Published:** 2021-03-18

**Authors:** Thais Correia Costa, Min Du, Karolina Batista Nascimento, Matheus Castilho Galvão, Javier Andrés Moreno Meneses, Erica Beatriz Schultz, Mateus Pies Gionbelli, Marcio de Souza Duarte

**Affiliations:** 1 Department of Animal Science, Universidade Federal de Viçosa, Viçosa MG 3657-000, Brazil; thais.correia@ufv.br; 2 Muscle Biology and Nutrigenomics Laboratory, Universidade Federal de Viçosa, Viçosa MG 3657-000, Brazil; 3 Department of Animal Sciences, Washington State University, Pullman, WA 99163, USA; min.du@wsu.edu; 4 Department of Animal Science, Universidade Federal de Lavras, Lavras MG 37200-900, Brazil; karolinanascimento@yahoo.com.br (K.B.N.); matheus.galvao@estudante.ufla.br (M.C.G.); ja.moreno.25@gmail.com (J.A.M.M.); mateus.pg@ufla.br (M.P.G.); 5 Department of Animal Science, Universidade Federal Rural do Rio de Janeiro, Seropédica RJ 23897-000, Brazil; ericabeatrizschultz@gmail.com

**Keywords:** beef cattle, dietary protein restriction, maternal effects, mid-gestation, skeletal muscle development

## Abstract

**Simple Summary:**

The intrauterine period plays a major role in skeletal muscle development and metabolism, including the formation of muscle fibers and adipose and connective tissue. Since the embryo and fetus depend on maternal nutrition to develop and grow, understanding the effects and finding potential strategies of skeletal muscle manipulation may be a valuable alternative to enhance beef cattle performance postnatally. In the present study, we evaluated the effects of maternal protein restriction during mid-gestation on the short and long -term skeletal muscle composition of the offspring. Our results suggest that the detrimental effects of maternal protein restriction during mid-gestation were associated with decrease in muscle fibers formation and may have contributed to the increase in collagen content in the skeletal muscle of the offspring. Although the changes in muscle fiber metabolism were not persistent, maternal protein restriction may contribute to such a short-term alteration. Our findings highlight the importance of an adequate nutritional plane for pregnant beef cows, to improve offspring’s performance, and consequently, the meat quality.

**Abstract:**

We aimed to investigate the effects of maternal protein restriction during mid-gestation on the skeletal muscle composition of the offspring. In the restriction treatment (RES, n = 9), cows were fed a basal diet, while in the control (CON, n = 9) group cows received the same RES diet plus the protein supplement during mid-gestation (100–200d). Samples of *Longissimus dorsi* muscle were collected from the offspring at 30d and 450d postnatal. Muscle fiber number was found to be decreased as a result of maternal protein restriction and persisted throughout the offspring’s life (*p* < 0.01). The collagen content was enhanced (*p* < 0.05) due to maternal protein restriction at 30d. *MHC2X* mRNA expression tended to be higher (*p* = 0.08) in RES 30d offspring, however, no difference (*p* > 0.05) was found among treatments at 450d. Taken together, our results suggest that maternal protein restriction during mid-gestation has major and persistent effects by reducing muscle fiber formation and may slightly increase collagen accumulation in the skeletal muscle of the offspring. Although maternal protein restriction may alter the muscle fiber metabolism by favoring the establishment of a predominant glycolytic metabolism, the postnatal environment may be a determinant factor that establishes the different proportion of muscle fiber types.

## 1. Introduction

In tropic regions, due to seasonal variations of dry and rainy period, beef cattle reproduction and lactation is synchronized with the rainy season to maximize the intake of high-quality forages. Nonetheless, in crucial stages of gestation, such as, mid-gestation, the quantity and quality of the pastures is limited [1], characterized by high insoluble fiber and lignin but low nitrogen content [2]. As the pregnancy advances, cows’ nutrient requirements increase, and the gravid uterus requires greater amounts of glucose and amino acids to foster fetal growth and development [3]. In a protein deficiency scenario, commonly experienced by the dams during mid-gestation, proteins are mobilized from maternal tissues (especially skeletal muscle) in an attempt to provide gluconeogenic precursors for glucose production needed to support fetal development [4]. 

Hence, strategies of maternal supplementation during critical periods of gestation [5] or offspring’s post-natal supplementation [6] are adopted in order to minimize the damages caused by maternal restriction during gestation. However, since the number of muscle fibers are fixed at birth, offspring’s supplementation may be limited in improving beef cattle potential for muscle growth in the postnatal stages of life. In addition to myogenesis, adipogenesis and fibrogenesis begin around mid-gestation as a competitive process since these cellular lineages are originated from a common ancestor cells, named mesenchymal stem cells [7]. Although adipogenesis is not limited to prenatal stages, its hyperplasia potential decreases over time [8]. Thus, maternal feed restriction directly affects fetal development by changing skeletal muscle composition, and ultimately impacts postnatal performance.

Meat quality is a multifactorial parameter, roughly affected by extrinsic and intrinsic factors. Among the factors affecting meat quality, the composition of muscle fibers [9], intramuscular fat (marbling level) [10] and connective tissue structure [11] may be, at least partially, manipulated during the prenatal stage in order to produce a desirable final body composition. Although many studies have reported the negative effects of maternal restriction during gestation on offspring skeletal muscle [12,13,14], there were no studies addressing long-term effect of maternal protein restriction during mid-gestation in beef cattle. Moreover, it remains unclear whether the negative changes can be partially rescued during postnatal stages. 

In the current study, we hypothesized that maternal protein restriction during mid-gestation would alter the skeletal muscle development in the offspring which would persist until the finishing phase. Thus, the skeletal muscle composition in two distinct times of offspring cattle was examined in order to monitor the impacts of mid-gestation nutrient deficiency on muscle growth of female and male calves.

## 2. Materials and Methods

### 2.1. Animal Husbandry, and Experimental Design

All animal care and handling procedures were approved by the Animal Care and Use Committee of the Department of Animal Science at the Universidade Federal de Lavras, Lavras, Minas Gerais, Brazil (protocol No. 015/17). Eighteen pregnant Tabapuã (*Bos indicus*) cows weighing 480.11 ± 82.97 kg and 5.69 ± 4.14 years of age (mean ± SD), gestating males (n = 8) and females (n = 10), were randomly assigned at day 80 of gestation into two treatments, according to the Scheme I. 

The cows were confined in individual pens (20 m^2^) and submitted to an adaptation period of 20 days. The treatments were applied from day 100 of gestation to day 200, representing the mid-gestation. Experimental diets were offered in the form of total mixture ration (TMR) and consisted in treatment control (CON; female = 4; male = 5), where the cows were fed a basal diet plus a protein supplement at the level of 0.35% of the body weight (3.5 g/kg/day); and treatment restricted (RES, female = 6; male = 3), where the cows were fed a basal diet ad libitum of roughage (corn silage and sugarcane bagasse) containing low crude protein (CP) and a high neutral detergent fiber (NDF) content. The protein supplement consisted of a 50:50 mixture of soybean meal and a commercial supplement (40% CP; Probeef Proteinado Sprint^®^, Cargill Nutrição Animal, Itapira, SP, Brazil). Table 1 contain the composition of the diets. 

From 200 days of gestation until parturition all the cows were fed ad libitum with corn silage (DM = 35.2%; CP = 7.2%, and NDF = 54.9%) and a commercial mineral mixture. The offspring were submitted to the same nutritional conditions after birth. At the weaning phase, all calves were kept with its dams in a high-quality tropical pasture (Brachiaria decumbens cv. Marandu; DM = 29.7 %; CP = 13.0 %; NDF = 62.6%) in an intensive grazing management. Weaning was performed 200 days after birth. After weaning, the animals were kept in pasture for 60 days to promote group uniformity. At 260 days of age, calves were assigned to feedlot with individual pens and fed with rising levels of concentrate until harvest (~450d). Heifers and young bulls had free access to water and were fed twice daily (0700 h and 0100 h). The finishing phase were performed from 390 to 450 days of age. The diet offered during the finishing phase (Table 1) was composed of corn silage (268 g/kg DM basis), ground corn (582 g/kg DM basis), and commercial supplement (150 g/kg DM basis), considering the Nutrient Requirements of Zebu and Crossbred Cattle (BR-CORTE) [15].

### 2.2. Skeletal Muscle Sampling

Samples of *Longissimus* muscle, located at the level of 10th and 11th ribs, were collected through biopsy from the calves at day 30 after birth and after feedlot period (~450d). Briefly, the *Longissimus dorsi* area was cleaned with 70% ethanol, and the incision was performed 10 min after local anesthesia treatment (Lidocaine 2%) approximately 3 cm^3^ of tissue was collected and part of tissue was snap frozen in liquid nitrogen and stored at −80 °C, and the other part was fixed in fresh 10% (wt/vol) buffered formalin for further histological analysis. The local of collection was sutured and the animals were accompanied and treated with antibiotics and anti-inflammatory drug. The sutures were removed two weeks after biopsy.

### 2.3. Morphometric Evaluation by Histochemical and Image Analysis

Skeletal muscle samples previously fixed in fresh 10% (wt/vol) buffered formalin in phosphate buffer was dehydrated using a crescent ethanol series and embedded in paraffin. Sections of 5 μm were obtained using a rotary microtome (RM 2265, Leica Biosystems, Nussloch, Germany) and stained with Masson’s trichrome [16]. For observation of the number and area of muscle fibers, ten digital images of muscle sections per animal were obtained at 40× magnification and analyzed using the ImageJ software (National Institute of Health, Baltimore, MD, USA). To measure intramuscular collagen content (stained in blue), images obtained at 20× magnification were converted into grayscale, threshold to the same extent to highlight and quantify the collagen area, which was expressed as the percentage of the total area [17].

### 2.4. Total RNA Extraction and mRNA Expression Analysis

The frozen samples were powdered in liquid nitrogen and total RNA extracted from 0.1 g of tissue using Trizol^®^ (InvitrogenTM, Thermo Fisher Scientific^®^, Hillsboro, OR, USA) using the manufacturer’s recommendations. Total RNA was quantified with a NanoDrop spectrophotometer. The RNA samples were reverse transcribed into cDNA using the GoScriptTM Reverse Transcription System Kit (Promega Corporation, Madison, WI, USA), and quantified [absorbance (A) ratio at 260 and 280  nm] using a NanoDrop spectrophotometer, with an optimal 260/280 ratio between 1.8 and 2.0. The primers of the genes (Table 2) were designed using PrimerQuest software (www.idtdna.com/Scitools/Applications/PrimerQuest; access date: 05/2020) with sequences obtained in GenBank (www.ncbi.nlm.nih.gov; access date: 05/2020).

Real-time quantitative PCR was performed in the thermal cycler ABI Prism 7300 Sequence Detection System (Applied Biosystems, Foster City, CA, USA) using the detection method SYBR Green (Applied Biosystems—Foster City, CA, USA) and GoTaq^®^ qPCR Master Mix kit (Promega Corporation, Madison, WI, USA) using the following cycle parameters: 95 °C for 2  min and 40 cycles at 95  °C for 15  s and 60  °C for 60  s. The results are expressed relative to 18S using ln(2 ^−ΔΔCt^ + 1) [18].

### 2.5. Statistical Analyses

Data from stage 1 representing the 30d old offspring were analyzed separately from data from stage 2 that represent 450d old offspring, through the model:

(1) $$Y_{ijk}=\mu +N_{i}+S_{j}+{\left(NS\right)}_{ij}+e_{ijk}$$

where, $Y_{ijk}$ is the observed measurement; μ is the overall mean; $N_{i}$ is the fixed-effect of the *i*th level of maternal nutrition; $S_{j}$ is the fixed-effect of the *j*th level of offspring sex; $NS_{ij}$ is the interaction between N and S; and the $e_{ijk}$ is the random error associated with $Y_{ij}$ with $e_{ijk}$~N(0,$\sigma_{e}^{2}$). Prior to the final analyses, extreme data were removed when Studentized residuals within ± 1.5 standard deviations, and normality (*p* > 0.05) was assessed using Shapiro–Wilk’s test. Least square means were estimated for all effects and compared using Tukey’s method adopting α= 0.05 of probability for type I error and between α = 0.05 and 0.10 of probability for trends. All statistical procedures were performed using the software R.

## 3. Results

### 3.1. Maternal Intake and Performance 

The protein supplementation (CON) between 100 and 200 days of gestation increased (*p* = 0.03) the cow’s total daily dry matter intake by 26% (7.44 vs. 5.88 kg/day) compared to RES cows. There was no effect of fetal sex (*p* = 0.89) or interaction between fetal sex and nutritional treatment (*p* = 0.20) on maternal feed intake. The diet consumed between 100 and 200 days of gestation met the equivalent of 83% and 104% of the energy requirements and 59% and 104% of the protein requirements of RES and CON cows, respectively (calculated according to the nutrient requirements for pregnant Zebu cows [19]). Protein supplemented (CON) cows had greater (*p* = 0.03) average daily gain (ADG) than RES cows (0.262 vs. −0.184 kg/d), as well as the cows pregnant with female calves which had greater (*p* = 0.04) ADG than cows pregnant with male calves. No interaction effect (*p* = 0.19) was observed for nutritional treatment and fetal sex on maternal ADG between 100 and 200 days of gestation. 

### 3.2. Muscle Cell Area, Number, and Collagen Content 

There was no interaction effect (*p* > 0.05) between maternal nutrition and sex regarding the histological variables, muscle cell area, number, and collagen content evaluated in the current study. 

**Figure 1:**
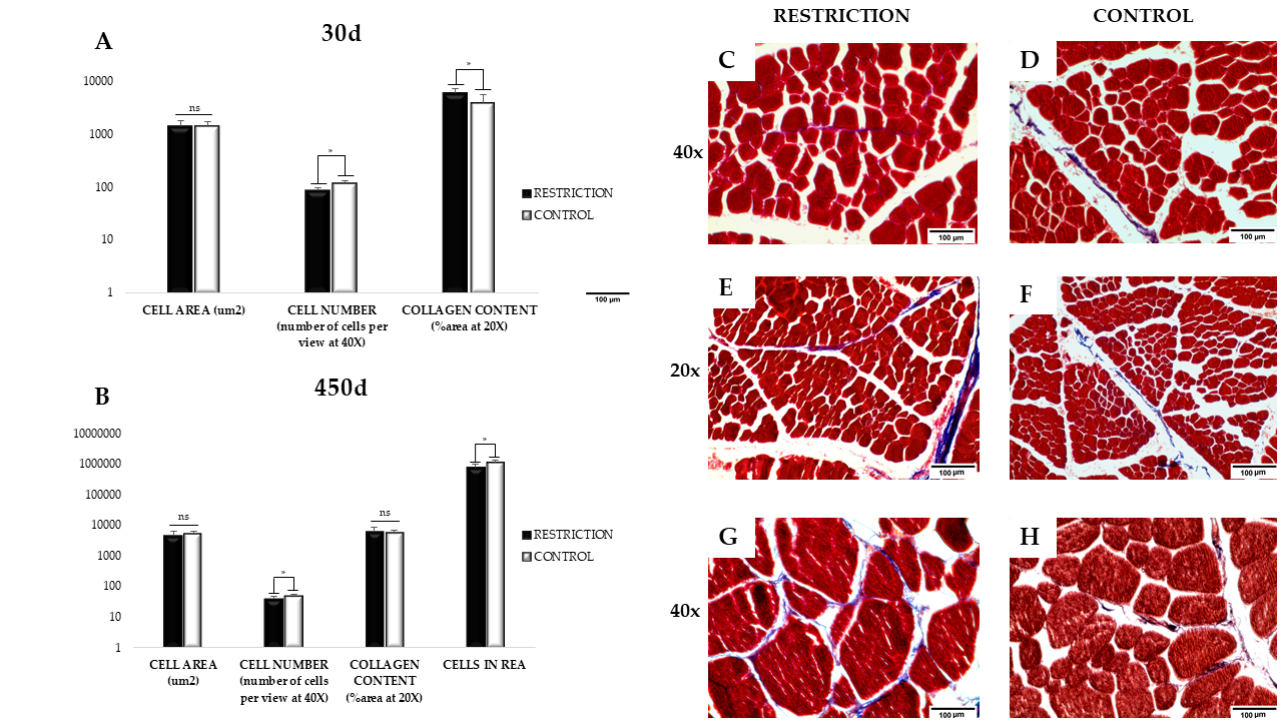
Cell area (um^2^), cell number (number/view), collagen content (%area) of the skeletal muscle of the offspring at 30d and 450d of age resulting from maternal protein restriction and control group (**A**,**B**), and number of muscle cells in rib eye area (REA) measured at 450d (**B**). Bars represent means ± SEM. Histological images from the skeletal muscle of the offspring from restriction (**C**,**E**) and control (**D**,**F**) group at 30d. Histological images from the skeletal muscle of the offspring from restriction (**G**) and control (**H**) group at 450d. To combine different measurement units into the graph, the data were transformed into log10.

There was no difference (*p* > 0.05) in muscle fiber area between treatments, in both stages evaluated (Figure 1). However, muscle fiber hyperplasia was impaired by maternal protein restriction during mid-gestation. The muscle fiber number was reduced in restricted calves at 30d (*p* < 0.01), which persisted to the finishing stage (450d; *p* < 0.01), as well as the number of fibers in the rib eye area (*p* = 0.02; Figure 1B). Moreover, the offspring cattle of protein restricted cows had higher (*p* < 0.01) collagen content at 30d, compared with the control group (Figure 1A). No difference (*p* > 0.05) in collagen content was observed in 450d old animals (Figure 1B). 

### 3.3. mRNA Expression of Adipogenic Markers

**Figure 2:**
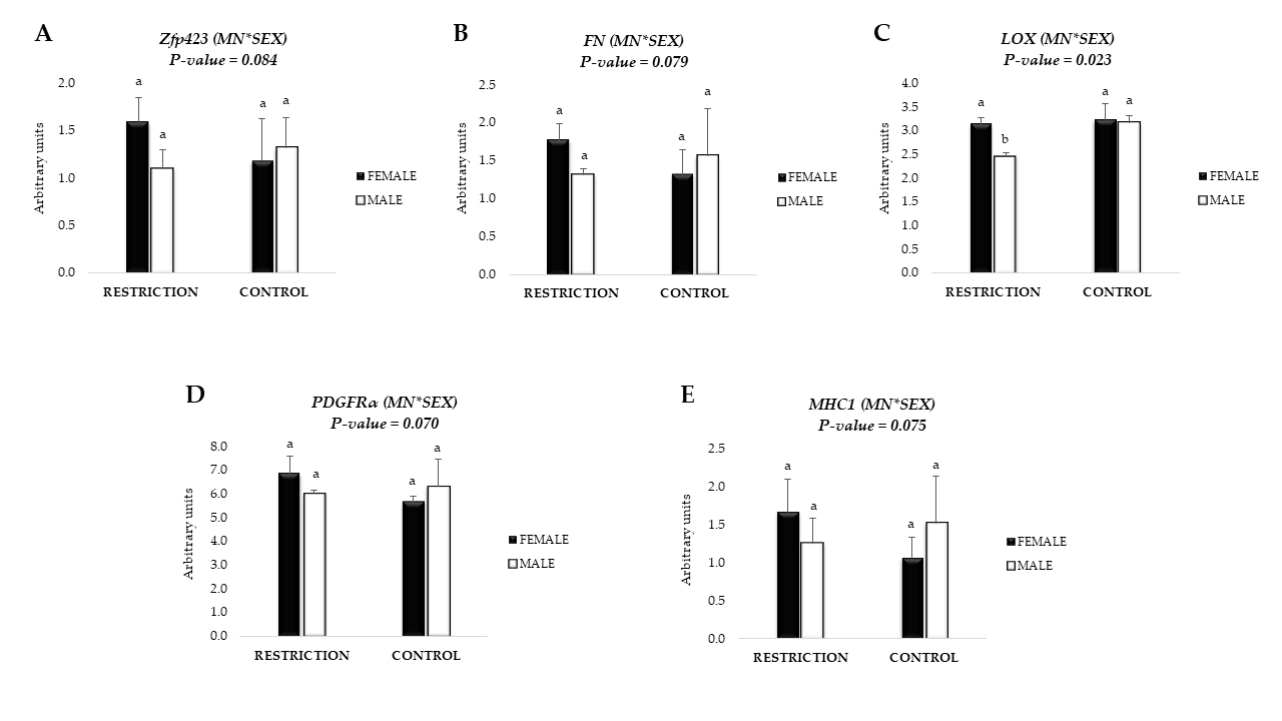
Interaction effect between maternal treatment (CON vs. RES) and offspring’s sex (FEMALE vs. MALE) on the relative gene expression of *Zfp423* (**A**), *FN* (**B**), *LOX* (**C**), *PDGFRα* (**D**), and *MHC1* (**E**) at 30d. Different letters indicate differences among groups. Tendency was considered when 0.10 < *p* > 0.05 and significant differences when *p* < 0.05.

For the expression of the early adipogenic marker *Zfp423* there was a tendency (*p =* 0.08) of an interaction effect between maternal nutrition and sex at 30d, however, males and females did not differ due to the maternal dietary treatments (Figure 2A).

The expression of the late adipogenic markers *CEBPα and PPARγ* did not differ between treatments in either stages evaluated (*p* > 0.05; Table 3 and Table 4).

### 3.4. mRNA Expression of Fibrogenic Markers and Collagen Content

There was a tendency (*p* = 0.08) of an interaction effect between maternal nutrition and sex at 30d regarding the expression of *FN*, however, males and females did not differ due to the maternal dietary treatments (Figure 2B). The expression of the fibrogenic related growth factor *TGFβ,* and the genes coding the components of the extracellular matrix (ECM), *COL1*, and *COL3* did not differ between treatments at 30d (*p* > 0.05; Table 3). 

**Figure 3:**
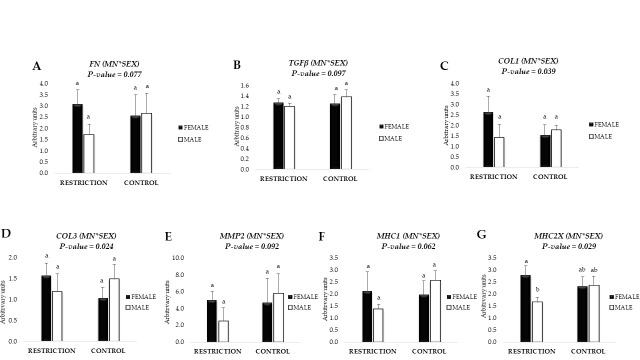
Interaction effect between maternal treatment (CON vs. RES) and offspring’s sex (FEMALE vs. MALE) on the relative gene expression of *FN* (**A**), *TGFβ* (**B**), *COL1* (**C**), *COL3* (**D**), and *MMP2* (**E**), *MHC1* (**F**), and *MHC2X* (**G**) at 450d. Different letters indicate differences among groups. Tendency was considered when 0.10 < *p* > 0.05 and significant differences when *p* < 0.05.

In the finishing phase (450d), there was a tendency of interaction between maternal nutrition and sex regarding the expression of *FN* (*p =* 0.08) and *TGFβ* (*p =* 0.10), however, males and females did not differ due to the maternal dietary treatments (Figure 3A,B). Furthermore, there was a significant interaction effect among maternal nutrition and sex regarding the expression of *COL1* (*p* = 0.04) and *COL3* (*p* = 0.02), without differences between females and males from both treatments (Figure 3C,D).

### 3.5. mRNA Expression of Markers related to Collagen Crosslinking and Remodeling

There was a significant interaction effect between maternal nutrition and sex regarding the *LOX* expression (*p* = 0.02) at 30d, where females from restriction group (RES) had higher expression of *LOX* compared with males from the same experimental group (Figure 2C). No difference was observed in the expression of *P4Ha1* (*p* = 0.41) between treatments at 30d (Table 3) In the finishing stage (450d), no differences (*p* > 0.05) were observed in the expression of the markers related with collagen crosslinking, *LOX* and *P4Ha1* (Table 4).

At 30d, there was no difference (*p* = 0.54) in the expression of the marker of collagen remodeling *MMP2* (Table 3). A tendency (*p* = 0.09) of interaction effect between maternal nutrition and sex was observed in the expression of *MMP2* at 450d, despite minor changes due to either treatment or sex (Figure 3E).

In both stages evaluated, there were no differences (*p* > 0.05) in the expression of the markers of collagen remodeling, *TIMP1*, and *TIMP2* between treatments (Table 3 and Table 4). 

### 3.6. mRNA Expression of Markers Related with Satellite Cell and Fibro-Adipogenic Progenitor (FAP) Cells 

The expression of *PAX7* tended (*p* = 0.10) to be higher in RES compared with CON at 30d (Table 3). In the finishing stage (450d), the expression of *PAX7* only differed between the sexes, where females had higher expression (*p* = 0.04) than males (Table 4).

There was a tendency (*p* = 0.07) of interaction effect between maternal nutrition and sex at 30d regarding the expression of *PDGFRα*, however, males and females did not differ due to the maternal dietary treatments (Figure 2D).

In the finishing phase (450d), there was no difference (*p* = 0.65) in the expression of *PDGFRα* between treatments (Table 4).

### 3.7. mRNA Expression of Myosin Heavy Chain Isoforms 

**Figure 4:**
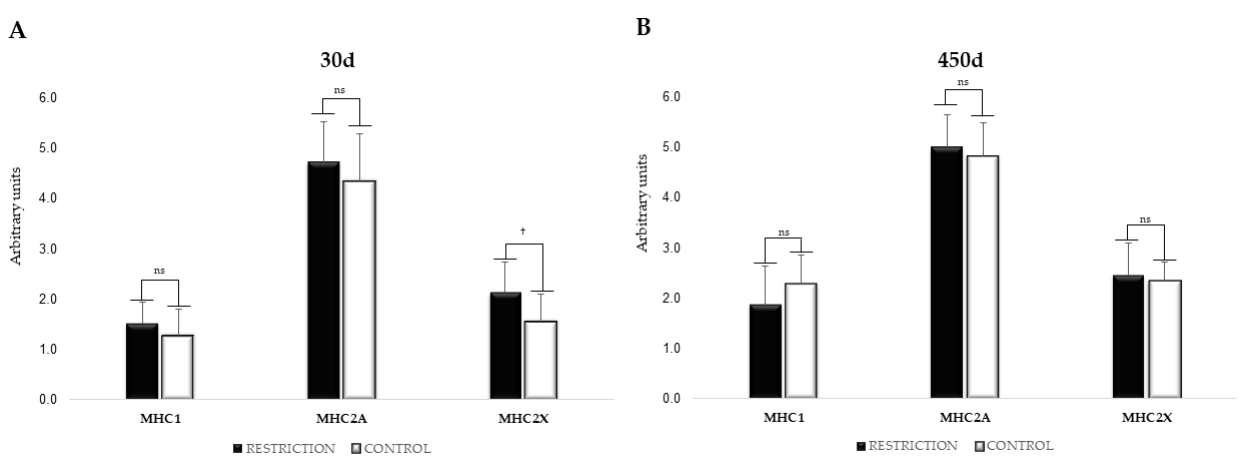
Relative gene expression (means ± SEM) of Myosin Heavy Chain (MHCs) isoforms in the skeletal muscle of the offspring resulting from maternal protein restriction and control group evaluated at 30d (**A**) and 450d (**B**); ns = not significant (*p* > 0.10); † tendency when 0.10 < *p* > 0.05.

There was a tendency in interaction effect between maternal nutrition and sex regarding the expression of *MHC1* (*p* = 0.08) at 30d, without differences between females and males from both treatments (Figure 2F). At 30d, the expression of *MHC2X* tended to be higher (*p* = 0.08) in RES compared with CON, while the expression of the other isoform, *MHC2A* did not differ between treatments (*p* = 0.40; Figure 4A). 

No differences (*p* = 0.60) between treatments were observed in the finishing stage, regarding the expression of *MHC2A* (Table 3; Figure 4B). There was a tendency (*p* = 0.06) in the interaction effect between maternal nutrition and sex regarding the expression of *MHC1* in finishing phase (450d), without differences between females and males from both treatments (Figure 3F). An interaction effect was also observed in the expression of *MHC2X* (*p* = 0.03) at 450d, where females from treatment RES presented higher expression of *MHC2X* than males, while in the CON group no differences were observed between females and males (Figure 3G).

## 4. Discussion

The meat industry demands high quality meats, seeking high marbled carcasses, which can be achieved by manipulating the skeletal muscle composition from prenatal stages to the postnatal life. Although it is well established that maternal restriction during gestation impairs the skeletal muscle development of the offspring [20,21,22], the mechanism and the long-term effects remain poorly studied. Through the evaluation of the skeletal muscle composition in two distinct stages of offspring’s life, the present study enabled the identification of differences in the number of muscle fibers, which were lower in the resulting offspring from maternal protein restriction during mid-gestation and this impairment persisted until the finishing phase. Consistent with previous studies, maternal restriction before the 210d of gestation has a major impact in muscle fibers number, due to myogenesis that begins during the embryonic stage (primary myofibers), persists in fetal stages (secondary myofibers), and slows until late gestation [7]. While maternal restriction after 210d of gestation may affect the muscle fiber size [23], which was not affected by the maternal dietary treatment in the current study. Although, muscle fiber number cannot increase after birth [24], a population of quiescent satellite cells may contribute to support postnatal growth and repair [25]. PAX7-positive cells are markers of satellite cells, which indicates the presence of undifferentiated myoblasts that have not reached their terminal differentiation [26]. Under the demand of hypertrophy, these cells may proliferate and fuse with existing muscle fibers [27]. Previous studies have shown that maternal feed restriction did not alter the expression of *PAX7* in goats [14], as well as the number of satellite cells in sheep [28]. A prenatal low protein diet resulted in reduced cell number, but the expression of *PAX7* and myogenic markers were unaltered in the skeletal muscle of young and adult rats [29]. In the current study, the expression of *PAX7* tended to be greater in RES at 30d of age, however, no difference was observed at 450d. Similarly, maternal undernutrition tended to increase *PAX7* expression in the offspring’s *Psoas major* muscle [30]. The inconsistency among studies may be due to the differences in the time of nutrient restriction. Satellite cells are formed during the late gestation [31], where no nutrient restriction was applied in our study. Another possible explanation is the diversity of cells that are able to express *PAX7*, such as vascular, immune [32], and central nervous system cells [33].

Besides muscle fiber formation, adipose and connective tissue are established during the prenatal period. The population of fibro-adipogenic progenitor (FAPs) cells holds the capacity of differentiate into adipocytes and fibroblasts, therefore, both processes may have antagonistic effects [34,35]. Marbling is a desirable meat quality parameter that depends on adipogenesis to enhance the number of intramuscular adipocytes. In cattle, adipogenesis and fibrogenesis initiate concomitantly with the secondary myogenesis during mid-gestation, however, most of adipocytes and fibroblast develops in late gestation [7]. The main transcriptional factor that regulates late adipogenesis, C/EBPα and PPARγ [36] play roles on targeting and enhancing the expression of genes responsible in adipocyte gene program [37]. Since the maternal protein restriction was applied during mid-gestation in the current study, adipogenesis might not be affected in the skeletal muscle of the offspring in both stages evaluated. 

Although, the expression of the cell surface marker *PDGFRα*, expressed by FAP cells [38,39], was not different between groups, fibrogenesis may be impaired in the skeletal muscle of the offspring. Fibroblasts are responsible for secreting components of connective tissue, including collagen and enzymes that catalyzes collagen cross-linking [31]. The process of collagen crosslink occurs slowly, increasing with age and contributing to the background toughness of meat [40]. The TGF-β signaling pathway is the most studied pro-fibrotic factor involved in tissue fibrosis [41,42]. After the binding of TGF-β to its receptor, the Smad complex translocates to the nucleus, recognizes the Smad-binding elements and initiates the transcription of the components of the extracellular matrix (ECM) [43], including *FN*, *COL1*, and *COL3*. At 30d the skeletal muscle of offspring resulting from maternal protein restriction, presented higher collagen content, despite the lack of difference in the expression of fibrogenic key genes. Collagen accumulation, in RES offspring at 30d may have occurred due to an adaptative mechanisms acquired in the restricted environment, which allowed the increase in this accumulation while in non-restricted conditions. Such adaptations include alteration in maternal metabolism and placenta in order to improve the efficiency of nutrient transfer to the fetus (for review, see [44]).

For further investigation of the mechanisms related to collagen content, we analyzed the key enzymes regulating collagen crosslinking and remodeling. Studies have shown that collagen content is positively correlated with collagen crosslinking and negatively correlated with collagen turnover [45]. Such correlation regarding collagen crosslinking was not observed in the present study, where the expression of the enzyme responsible for collagen crosslinking did not differ between treatments in addition to the expression of *LOX* that appears to be sex dependent at 30d animals. This could indicate that despite the accumulation, the crosslinking of collagen fibers was not altered as a result of maternal protein restriction. Collagen remodeling and consequently ECM homeostasis is mediated by the enzymes MMPs and TIMPs [46], both of which regulate collagen synthesis and degradation. MMPs degrade the majority of the ECM proteins [46,47], while the TIMPs inhibit the activity of MMPs [47], therefore balancing the tissue metabolism. The maternal restriction during the mid-gestation did not affect the overall collagen remodeling in the skeletal muscle of offspring at both stages evaluated, however the maternal treatment effect in the expression of fibrogenic markers in the skeletal muscle of the offspring at the finishing phase (450d) may be sex dependent. Moreover, the lack of visible differences in collagen content at 450d may have occured due to a partial inefficiency in collagen remodeling.

In response to the restricted intrauterine environment, not only may the cell commitment of the skeletal muscle components be altered, but also the use of energy sources. The relative proportion of muscle fiber types varies depending on species, muscle function, breed, gender, age, among others [48]. Type I fibers are characterized by the slow speed of contraction, and by the oxidative (aerobic) metabolism, which uses fatty acids as the main energy source [49]. Meanwhile, type II fibers are classified as fast speed contraction fibers, which use glucose as the main energy source [49]. In the current study, maternal protein restriction during mid-gestation tended to increase the expression of *MHC2X* in the skeletal muscle of the offspring at 30d, implying a predominantly glycolytic metabolism, however at 450d there were no differences in the expression of *MHC2X* compared to CON group. These data are consistent with previous findings [50,51,52], where maternal undernutrition contributed for the increase in type II muscle fibers in the skeletal muscle of the offspring. However adequate postnatal diet and environment allowed the fiber type switching to occur [28,50].

## 5. Conclusions

In conclusion, our results demonstrate that maternal protein restriction during mid-gestation impairs muscle fiber formation in the skeletal muscle of offspring, by decreasing the muscle fiber number and persisting until the finishing phase. Adipogenesis in the skeletal muscle of the offspring was not impaired by maternal protein restriction during mid-gestation, however, the enhancement of fibrogenesis may have an impact on collagen content accumulation accompanied by a non-altered and sex dependent collagen crosslink on 30d old offspring, in addition to an inefficient collagen remodeling at the finishing stage. Moreover, protein restriction of cows during mid-gestation may have a short-term effect on offspring’s muscle fiber metabolism, which does not have lasting effects on cattle at harvest.

## Figures and Tables

**Scheme I:**
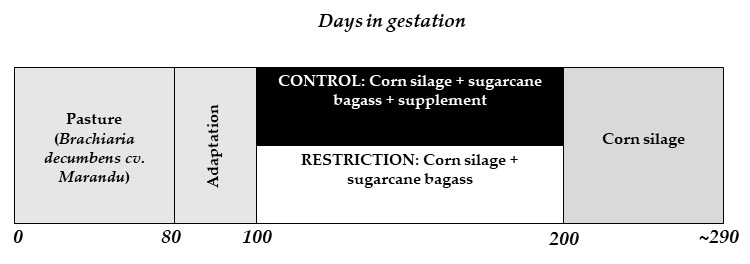
Scheme showing the period of the dietary treatments according to the gestational timeline.

**Table 1:** Chemical composition (mean) of the diets offered to the pregnant cow during mid-gestation and finishing diet offered to the offspring.

Item	Mid-Gestation ^1^		Offspring ^2^	
	Basal Diet	Supplement	Corn Silage	Concentrate
DM (g/Kg)	418	881	458	907
OM (g/Kg DM)	951	957	960	885
CP (g/Kg DM)	53.3	400	99.1	351
NDFap (g/Kg DM) ^3^	631	213	468	167
NFC (g/Kg DM)	242	342	367	340
EE (g/Kg DM)	24.1	41.2	25.8	25.5

^1^ Experiemtal diets were offered to pregnant cows from day 100 to 200 of gestation; ^2^ Diets offered to the offspring during the finishing phase; DM = dry matter; OM = organic matter; CP = crude protein; NDFap = neutral detergent fiber corrected for ash and protein; NCF = non-fiber carbohydrate; EE = ether extract; ^3^ NFC = 100 − (%NDF + %CP + %EE + %Ash).

**Table 2:** List of primers for relative gene expression by RT-qPCR.

Gene Name	Gene Abbreviation	Primer
Paired box 7	*PAX7*	F: CGGGCATGTTTAGCTGGGAGA
		R: TCTGAGCACTCGGCTAATCGAAC
Platelet-derived growth factor receptor A	*PDGFRα*	F: GCTCCCTTCCTTTGTCTCTTAC
		R: CGATTTGTCCTCCCATCTAACC
Myosin heavy chain 1	*MHC1*	F: ATCGCTGAATCCCAGGTCAA
		R: ACCAAGATGTGGCACGGCTA
Myosin heavy chain 2A	*MHC 2A*	F: CACCCTGGAGCAGACAGAGA
		R: TCCCTGGATTTGCGTGATG
Myosin heavy chain 2X	*MHC 2X*	F: TTTCCAGACCGTGTCTGCTC
		R: GGGATGATGCAGCGTACAAAG
Zinc finger protein 423	*ZFP423*	F: CCAGATGACCTTCGAGAATGAG
		R: CACTAGCTGTAGCAGGACAATAA
CCAAT-enhancer-binding protein alpha	*C/EBPα*	F: GGCAACGACTTTGACTACC
		R: CTCGTACAGAGGCTCCAG
Peroxissome proliferator activated-receptor gamma	*PPARγ*	F: TCCACTCCGCACTATGAG
		R: GGGATACAGGCTCCACTT
Transforming growth factor beta	*TGFβ*	F: AACCTGTGTTGCTCTCTCGG
		R: GAGGTAGCGCCAGGAATTGT
Fibronectin	*FN1*	F: CTGAGACCACCATCACCATTAG
		R: CTCGGAACTGTAAGGGTTCTTC
Collagen type I, alpha 1	*COL1A1*	F: CCACCCCAGCCGCAAAGAGT
		R: ACGCAGGTGACTGGTGGGATGTC
Collagen type III, alpha 1	*COL3A1*	F: GGCCCCCTGGAAAGGACGGA
		R: CCCCGCCAGCACCACAACAT
Lysyl oxidase	*LOX*	F: CAGAAGATCCAATGGGAGAACA
		R: TGGCATCAAGCAGGTCATAG
Prolyl 4-Hydroxylase Subunit Alpha 1	*P4HA1*	F: GGATGAGTGGGACAAGCCTC
		R: ACCGTCTCCAAGTCTCCTGT
Matrix metalloproteinase-2	*MMP2*	F: CGGCAAGTATGGCTTCTG
		R: CTCCTCCTGTGGGTCTTC
TIMP metallopeptidase inhibitor 1	*TIMP1*	F: CAGAGAGGCTACACCAGAG
		R: CACAACCAGCAGCATAGG
TIMP metallopeptidase inhibitor 2	*TIMP2*	F: GAAGGAGGTGGACTCTGG
		R: CCGGAGAGGAGATGTAGC
18 S ribosomal	*18S*	F: CCTGCGGCTTAATTTGACTC
		R: AACTAAGAACGGCCATGCAC

**Table 3:** Least square means ± standard errors for mRNA expression of adipogenic, fibrogenic, collagen crosslinking, collagen remodeling, and satellite cells markers evaluated on the skeletal muscle of the 30d offspring according to maternal nutrition and sex.

Gene	Maternal Nutrition ^1^		Sex		*p*—Value ^2^		
	Restriction	Control	Female	Male	MN	S	MN×S
mRNA expression of adipogenic markers (arbitrary units)							
*C/EBPα*	6.20 ± 1.52	5.95 ± 2.40	6.46 ± 1.36	5.57 ± 2.53	0.796	0.403	0.596
*PPARγ*	8.25 ± 1.04	7.43 ± 0.98	7.96 ± 1.13	7.69 ± 1.01	0.116	0.876	0.360
mRNA expression of fibrogenic markers (arbitrary units)							
*TGFβ*	1.36 ± 0.13	0.99 ± 0.21	1.02 ± 0.18	1.00 ± 0.16	0.629	0.970	0.472
*COL1*	0.92 ± 0.15	0.93 ± 0.20	0.97 ± 0.16	0.88 ± 0.20	0.920	0.358	0.120
*COL3*	1.28 ± 0.16	1.13 ± 0.38	1.19 ± 0.28	1.23 ± 0.30	0.300	0.618	0.135
mRNA expression of collagen crosslinking markers (arbitrary units)							
*P4Ha1*	1.83 ± 0.52	1.62 ± 0.65	1.70 ± 0.62	1.76 ± 0.47	0.411	0.690	0.256
mRNA expression of collagen remodeling markers (arbitrary units)							
*TIMP1*	8.17 ± 0.60	8.30 ± 0.65	8.32 ± 0.68	8.13 ± 0.52	0.670	0.496	0.181
*TIMP2*	3.05 ± 0.16	2.75 ± 0.53	3.02 ± 0.27	2.76 ± 0.52	0.132	0.278	0.405
*MMP2*	2.32 ± 1.25	2.72 ± 1.33	2.61 ± 1.27	2.42 ± 1.34	0.537	0.667	0.330
mRNA expression of satellite cells marker (arbitrary units)							
*PAX7*	5.55 ± 0.26 ^a^	5.23 ± 0.37 ^b^	5.44 ± 0.39	5.33 ± 0.29	0.099	0.623	0.501

^1^ RES = restriction and CON = control cows. ^2^ The main effects of maternal nutrition (MN), sex (S) and their interaction (MN×S); ^a,b^ Within a variable, means differences (*p* < 0.05) or tendency (*p*-value between 0.05 and 0.10).

**Table 4:** Least square means ± standard errors for mRNA expression of adipogenic, collagen crosslinking, collagen remodeling, satellite cells, and fibro-adipogenic progenitor cells marker evaluated on the skeletal muscle of the 450d offspring (finishing phase) according to maternal nutrition and sex.

Gene	Maternal Nutrition ^1^		Sex		*p*–Value ^2^		
	Restriction	Control	Female	Male	MN	S	MN×S
mRNA expression of adipogenic markers (arbitrary units)							
*ZFP423*	2.52 ± 0.57	2.68 ± 0.62	2.54 ± 0.50	2.67 ± 0.71	0.598	0.718	0.139
*C/EBPα*	8.39 ± 1.91	8.82 ± 3.61	8.84 ± 3.27	8.31 ± 2.30	0.762	0.658	0.353
*PPARγ*	9.64 ± 1.64	8.74 ± 1.25	9.64 ± 1.47	8.49 ± 1.21	0.208	0.167	0.134
mRNA expression of collagen crosslinking markers (arbitrary units)							
*LOX*	4.14 ± 0.84	4.25 ± 1.31	4.42 ± 1.17	3.92 ± 0.91	0.845	0.314	0.294
*P4Ha1*	2.51 ± 0.26	2.69 ± 0.38	2.60 ± 0.34	2.58 ± 0.30	0.289	0.696	0.156
mRNA expression of collagen remodeling markers (arbitrary units)							
*TIMP1*	6.64 ± 2.97	5.93 ± 2.65	6.57 ± 2.66	6.01 ± 3.02	0.634	0.808	0.723
*TIMP2*	3.33 ± 0.54	3.04 ± 0.64	3.41 ± 0.57	2.94 ± 0.55	0.310	0.162	0.205
mRNA expression of satellite cells marker (arbitrary units)							
*PAX7*	6.66 ± 1.02	6.46 ± 0.89	6.95 ± 0.85 ^a^	5.98 ± 0.76 ^b^	0.638	0.042	0.412
mRNA expression of fibro-adipogenic progenitor cells marker (arbitrary units)							
*PDGFRα*	6.61 ± 2.47	7.08 ± 1.51	6.75 ± 2.48	6.98 ± 1.35	0.649	0.901	0.433

^1^ RES = restriction and CON = control cows. ^2^ The main effects of maternal nutrition (MN), sex (S) and their interaction (MN×S); ^a,b^ Within a variable, means differences (*p* < 0.05) or tendency (*p*-value between 0.05 and 0.10).

## Data Availability

Data will be made available, upon reasonable request to the corresponding author.
